# Takotsubo Syndrome in Patients With Acute Coronary Syndrome or Myocarditis

**DOI:** 10.31083/RCM39562

**Published:** 2025-08-29

**Authors:** Manuel Martínez-Sellés

**Affiliations:** ^1^Cardiology Department, Hospital General Universitario Gregorio Marañón, Instituto de Investigación Sanitaria Gregorio Marañón, CIBERCV, 28007 Madrid, Spain; ^2^School of Biomedical and Health Sciences, Universidad Europea, 28670 Madrid, Spain; ^3^School of Medicine, Universidad Complutense, 28007 Madrid, Spain

**Keywords:** stress cardiomyopathy, Takotsubo syndrome, acute coronary syndrome, trigger

## Abstract

Stress cardiomyopathy/Takotsubo syndrome (TTS) is a transient cardiac condition characterized by sudden and reversible left ventricular dysfunction, typically triggered by emotional or physical stress. The international TTS (InterTAK) score predicts the probability of suffering from TTS. However, the diagnostic algorithm includes three mutually exclusive diagnoses: acute coronary syndrome (ACS), TTS, and acute infectious myocarditis. Thus, we propose to include the conditions in which TTS is associated with ACS or myocarditis. While TTS is commonly associated with non-ischemic stressors, recent evidence has indicated that TTS can be found in patients with ACS. Nonetheless, in some cases, ACS may trigger rather than exclude TTS. Additionally, TTS could also prompt plaque ruptures in coronary arteries. Meanwhile, infections and conditions that cause myocarditis can also produce physical stress that may trigger TTS. Furthermore, TTS has been reported after confirmed viral myocarditis. This opinion article explores the intricate relationships between (i) TTS and ACS, and (ii) TTS and myocarditis, delving into the related pathophysiologies and diagnostic challenges. However, further research is required to elucidate the mechanisms that link TTS with these conditions.

## 1. Introduction

Stress cardiomyopathy/Takotsubo syndrome (TTS) was first identified in Japan in the early 1990s [1], named after the Japanese term for an octopus trap due to the distinctive shape of the left ventricle during systole observed in affected patients. TTS is an acute reversible form of myocardial dysfunction. Typical TTS is triggered by physical or emotional stressful events and is characterized by a peculiar left ventricular apical hypokinesia/akinesia abnormalities extended beyond a single epicardial coronary artery distribution, basal hyperkinesia (“apical ballooning”) and absence of a “culprit” coronary artery obstruction [2]. Atypical presentations include absence of a stressful trigger, wall motion abnormalities in other areas of the left ventricle (including midventricular and basal regions or focal segments), and presence of coronary artery disease.

**Fig. 1.**
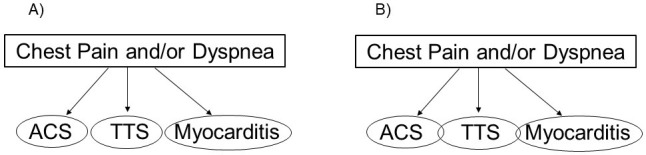
**Diagnostic algorithms**. (A) Current international takotsubo (InterTAK) diagnostic algorithm that includes three mutually exclusive diagnoses: acute coronary syndrome (ACS), takotsubo syndrome (TTS), and acute infectious myocarditis. (B) Our proposal to include conditions in which TTS is associated with ACS or myocarditis.

The International Takotsubo (InterTAK) diagnostic algorithm comprises seven parameters (female sex, emotional trigger, physical trigger, absence of ST-segment depression [except in lead aVR], psychiatric disorders, neurologic disorders, and QT prolongation) to obtain a predicted probability of suffering from TTS [3]. However, the current diagnostic algorithm includes three mutually exclusive diagnoses: acute coronary syndrome (ACS), TTS, and acute infectious myocarditis. We propose to include conditions in which TTS is associated with ACS or myocarditis (Fig. 1).

## 2. TTS and ACS

The first Mayo Clinic TTS diagnostic criteria from 2004 required the exclusion of obstructive coronary artery disease [4]. However, quickly it became clear that coronary artery disease frequently coexists with TTS [5]. Sharkey *et al*. [6] encountered 9 patients with features of both ACS and TTS among a population of 3506 consecutive ACS patients (0.3%) and 146 TTS patients (6.1%). Napp *et al*. [7] only found 3 patients with ACS among a total of 1016 TTS patients (0.3%), although the rate of obstructive coronary artery disease was much higher (23%) and 47 patients (4.6%) underwent percutaneous coronary intervention.

It is well-known that transient episodes of coronary ischemia may induce reversible left ventricular dysfunction. In fact, post-ischemic myocardial stunning is common after ACS/reperfusion and after other types of transitory myocardial ischemia (percutaneous coronary angioplasty, cardiac surgery, and ischemia induced by exercise, dobutamine or dipyridamole) [8]. In spite of being a strong physical and emotional stress factor, ACS is frequently regarded as an exclusion criterion for TTS. However, recent evidence shows that ACS and TTS may coexist and a growing body of case reports and case series show that TTS and ACS can occur concomitantly [9, 10, 11, 12, 13, 14, 15, 16, 17, 18, 19, 20, 21, 22, 23, 24, 25, 26, 27, 28, 29, 30, 31, 32, 33, 34, 35, 36, 37, 38, 39]. A myriad of emotional and physical stress factors have been reported to precipitate TTS. SCA is a major physical stress factor and frequently produces significant emotional impact [30]. It seems reasonable to assume that the high stress associated with ACS can also trigger TS. However, although ACS may trigger TTS, the opposite could also happen, as it has been hypothesized that the disrupted systolic motion of the left ventricular myocardium might promote the rupture of coronary artery plaques and coronary thrombotic occlusion [31]. This means that ACS may be the initial event, subsequently triggering TTS. Alternatively, TTS could precipitate ACS through plaque rupture mediated by this sudden change in left ventricular geometry and regional strain, but also due to the sympathetic discharge that causes vasoconstriction [7].

Differentiating the two entities or recognizing their concomitant occurrence might not straightforward, as there is considerable overlap in pathophysiological, clinical, and diagnostic characteristics. Moreover, emotional and physical triggering factors can occur both in TTS and in ACS, and, as we described above, one may precipitate the other. Perfusion-contraction mismatch might be the key to detect this double diagnosis [32], based on the fact that in patients with TTS, regional ventricular dysfunction typically does not adhere to the coronary anatomy, often extending beyond the boundaries of the supply region of the culprit artery. Moreover, cardiac magnetic resonance imaging frequently shows findings suggestive of myocardial infarction after TTS [33, 34] suggesting that the delayed enhancement seen in some patients may be a sign of a myocardial infarction that triggered TTS. We recognize that concomitant occurrence is unusual and that some authors prefer not to diagnose TTS in patients with ACS due to the fear of confusing clinicians and patients and the lack of therapeutic implications of the double diagnosis [35]. However, as Aristotle defined “to say of what is that it is, and of what is not that it is not, is true”.

## 3. TTS and Myocarditis

Infections and conditions that cause myocarditis can also produce physical stress that may trigger TTS and different authors have suggested myocarditis is common in TTS [36, 37, 38, 39, 40, 41]. Myocarditis is due to an inflammatory injury to the myocardium, typically due to viral infections, immune-mediated mechanisms, or toxins. TTS has been reported after confirmed viral myocarditis [42, 43, 44, 45], durvalumab-induced myocarditis [46], immune checkpoint inhibitor myocarditis [47] and lupus myopericarditis [48].

In addition, typical myocarditis magnetic resonance imaging results have been reported in patients with TTS [49, 50, 51, 52, 53, 54, 55, 56, 57]. Although magnetic resonance imaging in patients with TTS usually show myocardial edema without significant late gadolinium enhancement some patients also have the patchy or subepicardial late gadolinium enhancement suggestive of myocarditis. Moreover, endomyocardial biopsy, while not routinely performed in patients TTS, is sometimes done, and some biopsy results have shown inflammatory infiltrates typical of myocarditis [58, 59] instead of myocyte damage without inflammation (seen in pure TTS).

Thus, TC and myocarditis do not need to be mutually exclusive and three possibilities exist: (i) primary myocarditis triggering TTS, where inflammation and infection could lead to a catecholamine surge, a pathophysiology somewhat similar to pheochromocytoma [60, 61]; (ii) primary TTS with secondary inflammation, as TTS-induced myocardial injury may provoke localized inflammation, resulting in a myocarditis-like pattern [62, 63, 64, 65]; (iii) a common underlying trigger, where a shared external trigger (e.g., viral infection or systemic inflammation) causes simultaneous myocarditis and the physical stress that originates TTS [66, 67, 68, 69, 70]. Advances in cardiac magnetic resonance imaging and biopsy techniques have begun to reveal this underrecognized overlap. Greater awareness of the possibility of coexistence can lead to more accurate diagnosis, more personalized treatment, and better outcomes for patients presenting with this mixed acute myocardial dysfunction.

## 4. Discussion and Practical Considerations

TTS has traditionally been categorized as a distinct entity, often mutually exclusive from ACS and myocarditis. However, this rigid compartmentalization is increasingly challenged by clinical and imaging evidence showing significant overlap among these conditions. Current diagnostic algorithms frequently present TTS, ACS, and acute infectious myocarditis as separate paths, with criteria designed to rule out one in favor of the others. While useful for clarity, this approach oversimplifies complex clinical scenarios. In real-world settings, there are documented cases of co-occurrence of TTS triggered by ACS and myocarditis. By forcing clinicians to choose between ACS, TTS, and myocarditis, the diagnostic model may fail to capture the dynamic, multifactorial nature of myocardial injury in certain patients. This approach could have important implications for management, prognosis, and research. Recognizing and documenting the possibility of overlap in some patients could provide several benefits, allowing clinicians to better understand the mechanism of myocardial dysfunction in ambiguous presentations, changing treatment plans, and enabling more nuanced data collection and better understanding of pathophysiology. The risks of this more inclusive approach include overdiagnosis and an increase in resource utilization but the net clinical value of moving away from rigid diagnostic silos appears favorable. In some patients, especially those with atypical features or incomplete diagnostic clarity, it is reasonable to consider that TTS might coexist with ACS or myocarditis. The diagnostic algorithm must evolve to reflect the overlapping spectrum of myocardial injury syndromes, ensuring that care is guided by the complexity of individual presentations rather than artificial boundaries. For instance, in patients with ACS and electrocardiograms with QT prolongation [71] or high N-terminal pro–B-type natriuretic peptide/troponin ratio [72], coexistence with TS should be evaluated. In this regard, cardiac magnetic resonance imaging might be particularly useful to assess that possibility [73]. Magnetic resonance might also suggest the concurrence of TS in patients with myocarditis [63].

Most studies analyzed in this manuscript are case reports, case series or are limited by small sample sizes, and lack of methodological standardization. These limitations highlight the need for larger, multicenter prospective studies to clarify the frequence and clinical implications of TS in patients with ACS and myocarditis.

## 5. Conclusion

TTS might be seen in patients with ACS and in patients with acute myocarditis. Further research is needed to elucidate the pathophysiological mechanisms that explain these associations. Meanwhile we need to recognize that, in some uncommon cases, ACS may trigger rather than exclude TTS. In addition, TTS could prompt plaque ruptures in coronary arteries. TTS can also be associated with myocarditis. We propose that the diagnostic algorithm should include conditions in which TTS is seen together with ACS or myocarditis.

## References

[1] Sato H, Tateishi H, Uchida T, Dote K, Ishihara M, Sasaki K, Kodama K, Haze K, Hori M (1990). Takotsubo-type cardiomyopathy due to multivessel spasm. *Clinical Aspect of Myocardial Injury: From Ischemia to Heart Failure*.

[2] Crea F, Iannaccone G, La Vecchia G, Montone RA (2024). An update on the mechanisms of Takotsubo syndrome: “At the end an acute coronary syndrome”. *Journal of Molecular and Cellular Cardiology*.

[3] Ghadri JR, Wittstein IS, Prasad A, Sharkey S, Dote K, Akashi YJ (2018). International Expert Consensus Document on Takotsubo Syndrome (Part II): Diagnostic Workup, Outcome, and Management. *European Heart Journal*.

[4] Bybee KA, Prasad A, Barsness GW, Lerman A, Jaffe AS, Murphy JG (2004). Clinical characteristics and thrombolysis in myocardial infarction frame counts in women with transient left ventricular apical ballooning syndrome. *The American Journal of Cardiology*.

[5] Parodi G, Citro R, Bellandi B, Del Pace S, Rigo F, Marrani M (2013). Tako-tsubo cardiomyopathy and coronary artery disease: a possible association. *Coronary Artery Disease*.

[6] Sharkey SW, Kalra A, Henry TD, Smith TD, Pink VR, Lesser JR (2020). Coexistence of acute takotsubo syndrome and acute coronary syndrome. *Catheterization and Cardiovascular Interventions*.

[7] Napp LC, Cammann VL, Jaguszewski M, Szawan KA, Wischnewsky M, Gili S (2020). Coexistence and outcome of coronary artery disease in Takotsubo syndrome. *European Heart Journal*.

[8] Kloner RA, Arimie RB, Kay GL, Cannom D, Matthews R, Bhandari A (2001). Evidence for stunned myocardium in humans: a 2001 update. *Coronary Artery Disease*.

[9] Y-Hassan S (2015). Takotsubo syndrome triggered by acute coronary syndrome in a cohort of 20 patients: an often missed diagnosis. *International Journal of Cardiology Research*.

[10] Y-Hassan S (2014). Cardiac rupture in a patient with Takotsubo syndrome triggered by acute myocardial infarction: two messages. *International Journal of Cardiology*.

[11] Y-Hassan S (2015). Acute coronary syndrome or takotsubo syndrome: Most probably both of them, the first has triggered the second. *International Journal of Cardiology*.

[12] Y-Hassan S, Henareh L (2013). Spontaneous coronary artery dissection triggered post-ischemic myocardial stunning and takotsubo syndrome: two different names for the same condition. *Cardiovascular Revascularization Medicine*.

[13] Y-Hassan S, Jernberg T (2011). Bromocriptine-induced coronary spasm caused acute coronary syndrome, which triggered its own clinical twin-Takotsubo syndrome. *Cardiology*.

[14] Mencer N, Justice LT, Black W, Litton K (2019). A Rare Case of Takotsubo Syndrome and Acute Coronary Syndrome of the Right Coronary Artery. *Case Reports in Cardiology*.

[15] Espinoza Romero C, Correia VM, Lata W, De Paula Morales K, Valdivieso LB, Pin GO (2025). Takotsubo syndrome in the context of atherosclerotic acute myocardial infarction: the role of magnetic resonance imaging in determining cause or consequence. *Exploration of Cardiology*.

[16] Koeth O, Zeymer U, Schiele R, Zahn R (2010). Inferior ST-Elevation Myocardial Infarction Associated with Takotsubo Cardiomyopathy. *Case Reports in Medicine*.

[17] Gurlek C, van Es J, van der Burgh PH, Galjee MA, van Birgelen C (2007). Full pattern of transient apical ballooning of the left ventricle triggered by minor myocardial infarction. *Netherlands Heart Journal*.

[18] Huczek Z, Filipiak KJ, Kochman J, Roik M, Piatkowski R, Opolski G (2008). Are normal coronary arteries a typical feature of apical ballooning syndrome?. *The American Journal of Emergency Medicine*.

[19] Dietrich PL, Cieslik M, Cammann VL, Schneiter S, Meyer MR, Templin C (2022). The answer to the riddle: Multimodality imaging for diagnosing a double hit of acute coronary syndrome and takotsubo syndrome. *Cardiology Journal*.

[20] Redfors B, Råmunddal T, Shao Y, Omerovic E (2014). Takotsubo triggered by acute myocardial infarction: a common but overlooked syndrome?. *Journal of Geriatric Cardiology*.

[21] Tota F, Ruggiero M, Sassara M, Locuratolo N, Sublimi Saponetti L, Frasso G (2013). Subacute stent thrombosis and stress-induced cardiomyopathy: trigger or consequence?. *American Journal of Cardiovascular Disease*.

[22] Ferrara F, Baldi C, Malinconico M, Acri E, Cirillo A, Citro R (2016). Takotsubo cardiomyopathy after acute myocardial infarction: An unusual case of possible association. *European Heart Journal. Acute Cardiovascular Care*.

[23] Messas N, Caspar T, Jesel L, Hess S, Girardey M, Radulescu B (2016). Takotsubo cardiomyopathy triggered by ischemic injury: When lateralmyocardial infarction precipitate apical ballooning syndrome. *International Journal of Cardiology*.

[24] Hurtado Rendón IS, Alcivar D, Rodriguez-Escudero JP, Silver K (2018). Acute Myocardial Infarction and Stress Cardiomyopathy Are Not Mutually Exclusive. *The American Journal of Medicine*.

[25] Chaumont M, Blaimont M, Briki R, Unger P, Debbas N (2020). Acute Coronary Syndrome Mimicking Takotsubo Cardiomyopathy or Takotsubo Cardiomyopathy Mimicking Acute Coronary Syndrome?. *Case Reports in Cardiology*.

[26] González Alirangues P, Artiaga de la Barrera V, García Jiménez C, Ortiz Cortés C (2024). Takotsubo Syndrome Triggered by Acute Myocardial Infarction. *Cureus*.

[27] Kato K, Sakai Y, Ishibashi I, Kobayashi Y (2015). Mid-ventricular takotsubo cardiomyopathy preceding acute myocardial infarction. *The International Journal of Cardiovascular Imaging*.

[28] Christodoulidis G, Kundoor V, Kaluski E (2017). Stress Induced Cardiomyopathy Triggered by Acute Myocardial Infarction: A Case Series Challenging the Mayo Clinic Definition. *The American Journal of Case Reports*.

[29] Ezad S, McGee M, Boyle AJ (2019). Takotsubo Syndrome Associated with ST Elevation Myocardial Infarction. *Case Reports in Cardiology*.

[30] Nishikawa H, Honda S, Noguchi M, Sakai C, Harimoto K, Kawasaki T (2023). Takotsubo cardiomyopathy induced by acute coronary syndrome: A case report. *Journal of Cardiology Cases*.

[31] Madias JE (2025). Is takotsubo syndrome probably an acute coronary syndrome after all?. *Clinical Research in Cardiology: Official Journal of the German Cardiac Society*.

[32] Wagener M, Twerenbold R, Zellweger MJ, Haaf P (2023). Concurrent acute coronary and takotsubo syndrome - two in one; Commentary. *The International Journal of Cardiovascular Imaging*.

[33] Girolamo OC, Surikow SY, Ong GJ, Nguyen TH, Kucia AM, Chirkov YY (2022). TakoTsubo Syndrome: First an Acute Coronary Vasculitis and Then Prolonged Myocarditis?. *Reviews in Cardiovascular Medicine*.

[34] Kundapur D, Nosib S, Nosib S (2021). Takotsubo syndrome unmasking malignant coronary artery anomaly in a patient presenting with acute coronary syndrome: Expanding definitions of Takotsubo cardiomyopathy?. *BMJ Case Reports*.

[35] Madias JE (2020). How common is comorbid takotsubo syndrome in patients with acute coronary syndromes?. *Catheterization and Cardiovascular Interventions*.

[36] Yalta K, Ucar F, Yilmaztepe M, Zorkun C (2018). Takotsubo cardiomyopathy and acute coronary syndromes: Are they always mutually exclusive?. *Indian Heart Journal*.

[37] Y-Hassan S (2017). Why do you not call the condition takotsubo syndrome triggered by acute coronary ischemia?. *Echocardiography*.

[38] Svab S, Pasotti E, Moccetti T, Pedrazzini GB (2018). Tako-tsubo cardiomyopathy, acute coronary syndrome, or both?. *European Heart Journal*.

[39] Madias JE (2015). Takotsubo syndrome + coronary artery disease vs Takotsubo syndrome + acute coronary syndromes. *The Journal of Invasive Cardiology*.

[40] Serrano-Rosa MÁ, León-Zarceño E, Giglio C, Boix-Vilella S, Moreno-Tenas A, Pamies-Aubalat L (2021). Psychological State after an Acute Coronary Syndrome: Impact of Physical Limitations. *International Journal of Environmental Research and Public Health*.

[41] Madias JE (2015). Could Takotsubo Syndrome Trigger Type I Myocardial Infarction?. *The American Journal of Cardiology*.

[42] Napp LC, Ghadri JR, Bauersachs J, Templin C (2015). Acute coronary syndrome or Takotsubo cardiomyopathy: The suspect may not always be the culprit. *International Journal of Cardiology*.

[43] Eitel I, Behrendt F, Schindler K, Kivelitz D, Gutberlet M, Schuler G (2008). Differential diagnosis of suspected apical ballooning syndrome using contrast-enhanced magnetic resonance imaging. *European Heart Journal*.

[44] Muellerleile K, Lund G, Groth M, Barmeyer A, Sultan A, Heitzer T (2010). Delayed-enhancement magnetic resonance imaging in patients with clinically suspected stress cardiomyopathy (Tako-tsubo). *RoFo: Fortschritte Auf Dem Gebiete Der Rontgenstrahlen Und Der Nuklearmedizin*.

[45] Showkathali R, Ramoutar A (2014). Takotsubo cardiomyopathy and acute coronary syndrome–overlapping diagnoses will lead to confusion. *European Journal of Internal Medicine*.

[46] Y-Hassan S (2014). Myocarditis and takotsubo syndrome: are they mutually exclusive?. *International Journal of Cardiology*.

[47] Khalid N, Chhabra L (2016). Takotsubo Cardiomyopathy and Viral Myopericarditis: An Association Which Should be Considered in the Differential Diagnosis. *Angiology*.

[48] Chhabra L (2015). Myopericarditis and Takotsubo cardiomyopathy association. *International Journal of Cardiology*.

[49] Kawai S, Shimada T (2014). Inflammation in takotsubo cardiomyopathy? Inquiry from “Guidelines for Diagnosis and Treatment of Myocarditis (JCS 2009)”. *Journal of Cardiology*.

[50] Eitel I, Schuler G, Thiele H (2009). Myocarditis mimicking Takotsubo cardiomyopathy or Takotsubo cardiomyopathy with secondary inflammation?. *European Journal of Heart Failure*.

[51] Perrino C, Imbriaco M, Magliulo F, Ponsiglione A, Puglia M, Stabile E (2016). Tako-tsubo syndrome and myocarditis: Two sides of the same coin or same side for two different coins?. *International Journal of Cardiology*.

[52] Sala S, Peretto G, Gramegna M, Palmisano A, Villatore A, Vignale D (2020). Acute myocarditis presenting as a reverse Tako-Tsubo syndrome in a patient with SARS-CoV-2 respiratory infection. *European Heart Journal*.

[53] Bigalke B, Klingel K, May AE, Kandolf R, Gawaz MG (2007). Human herpesvirus 6 subtype A-associated myocarditis with ‘apical ballooning’. *The Canadian Journal of Cardiology*.

[54] Bahlmann E, Schneider C, Krause K, Pankuweit S, Härle T, Kuck KH (2007). Tako-Tsubo cardiomyopathy (apical ballooning) with parvovirus B19 genome in endomyocardial biopsy. *International Journal of Cardiology*.

[55] Ruggieri F, Cerri M, Beretta L (2014). Infective rhomboencephalitis and inverted Takotsubo: neurogenic-stunned myocardium or myocarditis?. *The American Journal of Emergency Medicine*.

[56] Saito S, Hontsu S, Hiraoka J, Yamanaka A, Fujioka N, Shimada D (2024). A Rare Case of Overlapping Durvalumab-induced Myositis, Takotsubo-like Morphological Changes Caused by Myocarditis, and Myasthenia Gravis. *Internal Medicine*.

[57] Norikane T, Mitamura K, Yamamoto Y, Takami Y, Fujimoto K, Noma T (2022). Immune checkpoint inhibitor myocarditis mimicking Takotsubo cardiomyopathy on MPI. *Journal of Nuclear Cardiology*.

[58] Chhabra L, Khalid N, Kluger J, Spodick DH (2014). Lupus myopericarditis as a preceding stressor for takotsubo cardiomyopathy. *Proceedings*.

[59] Karamitsos TD, Bull S, Ferreira V, Alp NJ, Neubauer S (2011). Acute myocarditis mimicking reverse Takotsubo cardiomyopathy [published Erratum in Circulation. 2011; 124: e335]. *Circulation*.

[60] Andò G, Trio O, de Gregorio C (2010). Transient left ventricular dysfunction in patients with neurovascular events. *Acute Cardiac Care*.

[61] Jorge C, Sargento L, Gato Varela M, Canas da Silva P, G Almeida A, Nunes Diogo A (2012). Takotsubo syndrome or acute myocarditis? The role of cardiac magnetic resonance imaging. *Portuguese Journal of Cardiology*.

[62] Rolf A, Nef HM, Möllmann H, Troidl C, Voss S, Conradi G (2009). Immunohistological basis of the late gadolinium enhancement phenomenon in tako-tsubo cardiomyopathy. *European Heart Journal*.

[63] Crosier R, Almatrooshi N, Chih S, Stadnick E, Naji KA, Mielniczuk L (2022). Use of Cardiac Magnetic Resonance Imaging to Distinguish Between Acute Myocarditis and Takotsubo Cardiomyopathy. *CJC Open*.

[64] Neil C, Nguyen TH, Kucia A, Crouch B, Sverdlov A, Chirkov Y (2012). Slowly resolving global myocardial inflammation/oedema in Tako-Tsubo cardiomyopathy: evidence from T2-weighted cardiac MRI. *Heart*.

[65] Maree AO, Witzke C, Holmvang G, Lewis GD, Jneid H, Reardon LB (2013). Gadolinium enhanced MRI in patients with left ventricular apical ballooning syndrome implicates myocarditis as an etiology. *World Journal of Cardiovascular Diseases*.

[66] Baker JT, Cury R, Hernandez-Suarez DF (2024). Myocarditis as a Possible Underlying Cause for Mid-Ventricular Takotsubo Cardiomyopathy: A Case Report. *Cureus*.

[67] Canan A, Van Woerkom RC, Rajiah PS (2022). Myocarditis Mimicking Stress-Induced (Takotsubo) Cardiomyopathy. *Texas Heart Institute Journal*.

[68] Taghavi S, Chenaghlou M, Mirtajaddini M, Naderi N, Amin A (2020). Takotsubo syndrome without major stress mimicking myocarditis. *Anatolian Journal of Cardiology*.

[69] (1986). Case records of the Massachusetts General Hospital. Weekly clinicopathological exercises. Case 18-1986. A 44-year-old woman with substernal pain and pulmonary edema after severe emotional stress. *The New England Journal of Medicine*.

[70] Caforio ALP, Tona F, Vinci A, Calabrese F, Ramondo A, Cacciavillani L (2009). Acute biopsy-proven lymphocytic myocarditis mimicking Takotsubo cardiomyopathy. *European Journal of Heart Failure*.

[71] Margulescu AD, Premawardhana DA, Thomas DE (2025). Prevalence and severity of QT prolongation and other ECG abnormalities in takotsubo syndrome. *Journal of Electrocardiology*.

[72] Kilaru P, Kilaru P, Garikipaty S (2025). The role of cardiac biomarkers in evaluating takotsubo cardiomyopathy: a systematic review. *Cureus*.

[73] Uchida Y, Suzuki M, Hashimura H, Eizawa H (2025). Coexistence of myocardial infarction, spontaneous coronary artery dissection, and Takotsubo syndrome: a case evaluated by intravascular ultrasound and cardiac magnetic resonance imaging. *European Heart Journal. Case Reports*.

